# Regulation of the Two-Component Regulator CpxR on Aminoglycosides and β-lactams Resistance in *Salmonella enterica* serovar Typhimurium

**DOI:** 10.3389/fmicb.2016.00604

**Published:** 2016-04-27

**Authors:** Hui Huang, Yawei Sun, Li Yuan, Yushan Pan, Yanlin Gao, Caihui Ma, Gongzheng Hu

**Affiliations:** ^1^College of Veterinary Medicine, Henan Agricultural UniversityZhengzhou, China; ^2^College of Animal Science and Technology, Henan Institute of Science and TechnologyXinxiang, China; ^3^Animal Husbandry Bureau of Henan ProvinceZhengzhou, China

**Keywords:** *S. enterica* serovar Typhimurium, CpxR, aminoglycosides, β-lactams, resistance, AcrD

## Abstract

The two-component signal transduction system CpxAR is especially widespread in Gram-negative bacteria. It has been reported that CpxAR contributes to the multidrug resistance (MDR) in *Escherichia coli*. CpxR is a response regulator in the two-component CpxAR system. The aim of this study was to explore the role of *cpxR* in the MDR of *S. enterica* serovar Typhimurium. The minimal inhibitory concentrations (MICs) of various antibiotics commonly used in veterinary medicine for strains JS (a multidrug-susceptible standard strain of *S. enterica* serovar Typhimurium), JSΔ*cpxR*, JSΔ*cpxR*/p*cpxR*, JSΔ*cpxR*/pcpxR^*^, JSΔ*cpxR*Δ*acrB*, JSΔ*cpxR*Δ*acrB*/p*cpxR*, JSΔ*cpxR*Δ*acrB*/pcpxR^*^, 9 *S. enterica* serovar Typhimurium isolates (SH1–9), and SH1–9Δ*cpxR* were determined by the 2-fold broth microdilution method. The relative mRNA expression levels of *ompF, ompC, ompW, ompD, tolC, acrB, acrD, acrF, mdtA, marA*, and *soxS* in strains JS, JSΔ*cpxR*, and JSΔ*cpxR*/p*cpxR* were detected by real-time PCR. The results showed 2- to 4-fold decreases in the MICs of amikacin (AMK), gentamycin (GEN), apramycin (APR), neomycin (NEO), ceftriaxone (CRO), ceftiofur (CEF), and cefquinome (CEQ) for strain JSΔ*cpxR*, as compared to those for the parental strain JS. Likewise, SH1–9Δ*cpxR* were found to have 2- to 8-fold reduction in resistance to the above antibiotics, except for NEO, as compared to their parental strains SH1–9. Furthermore, 2- to 4-fold further decreases in the MICs of AMK, GEN, APR, and CEF for strain JSΔ*cpxR*Δ*acrB* were observed, as compared to those for strain JSΔ*acrB*. In addition, CpxR overproduction in strain JSΔ*cpxR* led to significant decreases in the mRNA expression levels of *ompF, ompC, ompW, ompD, tolC, acrB, marA*, and *soxS*, and significant increases in those of *stm3031* and *stm1530*. Notably, after all strains were induced simultaneously by GEN to the 15th passage at subinhibitory concentrations, strain JSΔ*cpxR*/p*cpxR* showed significant increases in mRNA expression levels of the efflux pump *acrD* and *mdtA* genes, as compared to strain JSΔ*cpxR*. Our results indicate that the two-component regulator CpxR contributes to resistance of *S. enterica* serovar Typhimurium to aminoglycosides and β-lactams by influencing the expression level of the MDR-related genes.

## Introduction

*Salmonella enterica* serovar Typhimurium is a food-borne pathogen that causes gastroenteritis in humans (Scherer and Miller, 2001) and fowl typhoid in poultry (Barrow et al., 2004). The prevalence of multidrug-resistant (MDR) *S. enterica* species in many parts of the world has become a significant public health concern. Drug resistance in many cases is attributable to synergy between reduced drug intake (mainly due to low outer membrane permeability) (Pagès et al., 2008; Li and Nikaido, 2009) and active drug export (via efflux pumps) (Zgurskaya and Nikaido, 2000; Pagès et al., 2010). Resistance nodulation-cell division (RND)-family efflux systems (including AcrAB, AcrAD, AcrEF, MdtEF, and MdtABC) are especially effective in generating resistance in Gram negative bacteria (Nikaido, 1996) and often have a wide substrate specificity (Nikaido and Pagès, 2012). In *Escherichia coli*, all five RND-family drug exporters confer resistance to β-lactam antibiotics (Nishino et al., 2003), and *acrD* is also known to participate in the efflux of aminoglycosides (Rosenberg et al., 2000; Nishino and Yamaguchi, 2001a; Aires and Nikaido, 2005; Nishino et al., 2007). Some outer membrane proteins, especially OmpF, OmpC, and OmpW, have been shown to contribute to antibiotic resistance in *E. coli* and *Salmonella* typhimurium (Nikaido, 2003). In addition, OmpD, STM3031, and STM1530 are associated with ceftriaxone (CRO) resistance in *S. enterica* serovar Typhimurium (Hu et al., 2011), and OmpW participates in resistance to neomycin (NEO) and ampicillin (AMP) in *E. coli* (Wu et al., 2012).

Two-component signal transduction systems (TCSs) are key in the sensory response of bacteria (Parkinson and Kofoid, 1992). Studies have elucidated that the TCSs EvgA and BaeR contribute to MDR by modulating production of the multidrug transporter in *E. coli* (Nishino and Yamaguchi, 2001b; Baranova and Nikaido, 2002; Nishino and Yamaguchi, 2002). The Cpx envelope stress response is controlled by a TCS consisting of the membrane localized sensor kinase CpxA and the regulator CpxR. CpxR mediates the output response as a transcriptional regulator through phosphorylation of its receiver domain with an aspartate (D51) moiety (Stephenson and Hoch, 2002; MacRitchie et al., 2008). Phosphorylated CpxR (CpxR-P), which functions as a transcription factor, activates and, in a small number of cases, represses transcription of target genes by binding to the promoter of target genes at the consensus sequence 5′-GTAAAN_5_GTAAA-3′ (De Wulf et al., 2002; Price and Raivio, 2009). In addition, the response regulator CpxR is also activated by some signals without the involvement of CpxA. For example, some cytoplasmic or growth signals, as well as excess carbon (glucose or pyruvate) in growth medium both activate CpxR independently of CpxA (Cuny et al., 2007; Wolfe et al., 2008).

In recent years, the CpxAR two-component system conferring resistance to antibacterial agent has received special attention. In *E. coli*, CpxR overproduction was found to confer resistance to β-lactams in an *acrB*-free background (Hirakawa et al., 2003a). CpxR-P also confers resistance to fosfomycin by directly repressing the expression of two genes, *glpT* and *uhpT*, in the enterohemorrhagic *E. coli* (EHEC) strain O157:H7 (Kurabayashi et al., 2014). Moreover, the CpxAR pathway contributes to *E. coli* resistance to antimicrobial peptides, such as ApoEdpl-W, polymyxin B, and melittin (Audrain et al., 2013) and protamine (Weatherspoon-Griffin et al., 2014). In *Klebsiella pneumoniae*, CpxR was able to directly bind to the promoter regions of *ompC*^*KP*^and *kpnEF*, which contribute to the *K. Pneumoniae* MDR phenotype (Srinivasan et al., 2012; Srinivasan and Rajamohan, 2013). In *S. enterica* serovar Typhimurium, studies about the effect of *cpxR* on the resistance are still very limited, only few reports showed CpxAR confers resistance to CRO (Hu et al., 2011) and the antimicrobial peptides protamine, magainin, and melittin (Weatherspoon-Griffin et al., 2011). However, whether CpxAR plays a role in resistance of S. *enterica* serovar Typhimurium especially clinical isolates to aminoglycosides and β-lactams and the molecular mechanisms underlying resistance to aminoglycosides and β-lactams remain unknown. In this study, we systematically investigated the role of *cpxR* in aminoglycoside and β-lactam resistance in both susceptible strains and clinical isolates of *S. enterica* serovar Typhimurium, and also explored the molecular mechanisms of CpxAR that confer resistance to aminoglycosides and β-lactams.

## Materials and methods

### Bacterial strains, plasmids, and bacteriophage

The bacterial strains, plasmids, and bacteriophage used in this study are listed in Table 1. *Salmonella enterica* serovar Typhimurium strain CVCC541, a clinical susceptible strain isolated from chicken in Changchun City, China, was supplied by the China Institute of Veterinary Drug Control (Beijing, China) and designated as JS in this report. Strains JSΔ*cpxR* and JSΔ*acrB* were generated from JS using the one-step inactivation of chromosomal genes method. Strain JSΔ*acrB*Δ*cpxR* was constructed by the phage P22-mediated transduction method using strain JSΔ*cpxR* as the donor and JSΔ*acrB* as the recipient. In this study, nine *S. enterica* serovar Typhimurium isolates were isolated from chickens collected from nine different regions of Henan province in China and named SH1–9.

**Table 1:** **Bacterial strains, plasmids, and phage used in this study**.

**Strain, plasmid, or phage**	**Relevant characteristics**	**References or source**
**STRAINS**		
JS	*S. enterica* Serovar Typhimurium CVCC541	Supplied by China Institute of Veterinary Drug Control
JSΔ*cpxR*	Derivative of JS that lacks *cpxR*	Huang et al., 2015
JSΔ*acrB*	Derivative of JS that lacks *acrB*	Huang et al., 2016
JSΔ*acrB*Δ*cpxR*	Derivative of JS that lacks both *cpxR* and *acrB*, Δ*cpxR::kan*	Huang et al., 2016
SH(1–9)	Clinical isolates from chicken in Henan province in China	This study
SH(1–9) Δ*cpxR*	Derivative of SH(1–9) that lack *cpxR*, Δ*cpxR::kan*	This study
**PLASMIDS**		
pKD4	Gene knowout help vector: rep_R6K_γ Ap^R^ FRT Km^R^ FRT	From *E. coli* Genetic Stock Center in Yale University
pKD46	Gene knowout help vector: ${\text{rep}}_{\text{pSC101}}^{\text{ts}}$ Ap^R^ P_araBAD_ γ β exo	
pBAD	Expression vector: rep_pBR322_Ap^R^ araC P_BAD_	Invitrogen Corporation
pBAD-CpxR	cpxR gene cloned to pBAD; Ap^R^	This study
pBAD-CpxR^*^	Mutation sequence CpxR^*^ cloned to pBAD; Ap^*R*^	This study
**PHAGE**		
P22HT105/int	Transduction medium of *Salmonella*	Supplied by Microbial Genomics Research Center of Harbin Medical University

### Construction of the expression plasmids pBAD-CpxR and pBAD-CpxR^*^

The complete open reading frame of *cpxR* was amplified by PCR with primers *Xhol*I-*cpxR*-F/*Hin*dIII*-cpxR*-R (Table 2) from the genomic DNA of strain JS. The mutation sequence cpxR^*^, which encodes a CpxR variant with an alanine residue at position 51 in place of aspartate, was engineered by overlapping PCR (Urban et al., 1997; Huang et al., 1999). The mutation site was generated through the design of primers Fm and Rm (Table 2). Three PCR reactions were performed to obtain the mutation sequence cpxR^*^. Primers *Xho*lI-*cpxR*-F/Rm were used for amplification of the anterior segment of *cpxR*, primers Fm/*Hind*III*-cpxR*-R were used for amplification of the second part of *cpxR*, and the primers *Xhol*I-*cpxR*-F/*Hind*III*-cpxR*-R were used for splicing by overlap extension PCR. Finally, the expression plasmids pBAD-CpxR and pBAD-CpxR^*^ were generated by inserting the target fragment to the multiple cloning site of vector pBAD. The expression level of target proteins were determined according to the concentration of the inducer L-arabinose (Guzman et al., 1995).

**Table 2:** **Sequences of primers used in this study**.

**Function**	**Primer**	**Sequence (5′ → 3′)**	**References or source**
Amplification of *cpxR* gene	*Xhol*I-*cpxR*-F *Hind*III*-cp*x*R*-R	CGCTCGAGATGAATAAAATCCTGTTAGT GCAAGCTTTCATGAAGCGGAAACCATCA	This study
Preparation of cpxR^*^	*Xhol*I-*cpxR*-F Rm	CGCTCGAGATGAATAAAATCCTGTTAGT ACTTTTGCTTGCCGTCATGATGCCGAAG	This study
	Fm *Hind* III*-cpxR*-R	CTTCGGCATCATGACGGCAAGCAAAAGT GCAAGCTTTCATGAAGCGGAAACCATCA	This study
**REAL-TIME RELATIVE QUANTITATIVE PCR**			
*ompF*	*ompF*-F *ompF*-R	CCTGGCAGCGGTGATCC AAATTTCTGCTGCGTTTGCG	Tatavarthy and Cannons, 2010
*ompC*	*ompC*-F *ompC*-R	TCGCAGCCTGCTGAACCAGAAC ACGGGTTGCGTTATAGGTCTGAG	Hu et al., 2011
*ompD*	*ompD*-F *ompD*-R	GCAACCGTACTGAAAGCCAGGG GCCAAAGAAGTCAGTGTTACGGT	Hu et al., 2011
*ompW*	*ompW*-F *ompW*-R	CAGCAGCAAAGTGCGTCCTTATGT AGACAGAGGCGCCAATTAACCAGT	Hu et al., 2011
*stm3031*	*stm3031*-F *stm3031*-R	TGCAAGCAGGGAGTAATAACGGGT TCACTTGGATACGCCCAGTCCCAT	Hu et al., 2011
*stm1530*	*stm1530*-F *stm1530*-R	CGTCTCGGTTTTGCTGGTTTGG GCCGTCATTTTTACCCTGATACTGC	Hu et al., 2011
*acrB*	*acrB*-F *acrB*-R	CGTGAGCGTTGAGAAGTCCT GGCGTCAGTTGGTATTTGGT	Li et al., 2009
*acrD*	*acrD*-F *acrD*-R	TCCGGCCAAATTGAATAGTT TCGGAACCGTCCTGATTAAC	Eaves et al., 2004
*acrF*	*acrF*-F *acrF*-R	TATCTGGCTGGATGCGAATCTGCT ACTTTGCCGAACTCTTCCGGATCT	Eaves et al., 2004
*mdtA*	*mdtA*-F *mdtA*-R	GAATGCGCGTCGTGATCTG TCCAGTTCCTGACGGGAAAC	Nishino et al., 2007
*marA*	*marA*-F *marA*-R	ATACATCCGCAGCCGTAAAA GTGATTCGCCATGCATATTG	Li et al., 2009
*soxS*	*soxS*-F *soxS*-R	TACGGTAACGCATCAAACA ACAGGCGGTGACGGTAAT	Li et al., 2009
16SrRNA	16SrRNA-F 16SrRNA-R	TTAGATACCCTGGTAGTCCACGC TTGCGGGACTTAACCCAAC	Li et al., 2009

*The underlined bases are restriction sites*.

### Construction of *cpxR*-deficient mutants of *S. enterica* serovar typhimurium isolates

The generation of strain JSΔ*cpxR::kan* was described in our previous study (Huang et al., 2015). The deletions were then transferred to nine *S. enterica* serovar Typhimurium isolates (SH1–9) by P22HT105/int transductions as previously described (Davis et al., 1980; Mann and Slauch, 1997). Nine *cpxR*-deficient mutants were designated as SH1–9Δ*cpxR* in this study.

### Antibiotic susceptibility testing

The minimal inhibitory concentrations (MICs) of selected antibiotics for all strains were determined by the 2-fold broth microdilution method according to the CLSI guidelines (Clinical and Laboratory Standards Institute, 2008, 2012). The antibiotics used for susceptibility determination were gentamycin (GEN), amikacin (AMK), apramycin (APR), NEO, CRO, ceftiofur (CEF), CEQ. *E. coli* ATCC 25922 was used for quality control in all susceptibility tests. All tests were performed independently at least three times.

### GEN induction testing

A single colony of each tested strain (JS, JSΔ*cpxR*, and JSΔ*cpxR*/p*cpxR*) was cultured in Luria-Bertani (LB) medium containing a 50% MIC of GEN at 37°C for 18 h. After growth overnight at 37°C, the cultures was diluted 1:100 in LB medium and cultured at 37°C for 18 h, and simultaneously the inducer GEN was added at subinhibitory concentrations. GEN induction testing of the strains was performed for 15 generations in this way.

### Expression levels of MDR-related genes

Total RNA was isolated from bacterial cultures using the MiniBEST Universal RNA Extraction Kit (TaKaRa Bio, Inc. Shiga, Japan) according to the manufacturer's instructions. OD260/OD280 values of total RNA were detected using a trace nucleic acid protein analyzer spectrophotometer (NanoDrop; Thermo Fisher Scientific, Waltham, MA, USA). Bulk cDNA samples were synthesized from total RNA using the PrimeScript™RT reagent Kit with gDNA Eraser (TaKaRa Bio, Inc.). The synthesized cDNA was confirmed by PCR and stored at –20°C until used. Real-time PCR was performed using the LightCycler®480 System (Roche Diagnostics, Indianapolis, IN, USA) with specific primer pairs (Table 2), cDNA template, and TaKaRa SYBR Premix Ex Taq II (TaKaRa Bio, Inc.). The 16S rRNA gene was chosen as a housekeeping gene. To precisely test the relative expression level of the genes of interest, standard curves of the amplification of all detected genes were individually established. CT values tested came within the linearity range for PCR amplification. Each sample was independently run at least twice. The 2^−Δ(ΔCT)^ method was used to calculate altered folds of the gene tested in the mutants, as compared to that in JS. Three independent experiments were performed under the same conditions.

### Statistical analysis

Statistical analysis was performed using SPSS version 17.0 software (IBM-SPSS, Inc., Chicago, IL, USA). Data were compared using the Student's *t*-test. A probability (*p*) value of > 0.05 was considered statistically significant.

## Results

### Deletion of *cpxR* increases susceptibility of JS to aminoglycosides and β-lactams

To examine whether the response regulator CpxR contributes to the drug resistance of *S. enterica* serovar Typhimurium, a *cpxR* deletion mutant, JSΔ*cpxR*, was generated from strain JS, and the complementary strain JSΔ*cpxR*/p*cpxR* was prepared through the introduction of the expression plasmid pBAD-CpxR into JSΔ*cpxR*. The MICs of a number of antibiotics for strain JS and JSΔ*cpxR* were then determined. As shown in Table 3, strain JSΔ*cpxR* showed 2–4-fold decreases in the MICs of GEN, AMK, APR, NEO, CRO, CEF, and CEQ, as compared to the parental strain JS. The MICs of the above antibiotics increased by 4-fold for the complementary strain JSΔ*cpxR*/p*cpxR*, as compared to those for JSΔ*cpxR*. These results clearly suggest that *cpxR* plays an important role in conferring resistance of *S. enterica* serovar Typhimurium to aminoglycosides and β-lactams. In addition, JSΔ*cpxR/*pcpxR^*^exhibited the same susceptibility as JSΔ*cpxR* to the tested antibiotics except for CEQ, which demonstrates that the susceptibility changes of *S. enterica* serovar Typhimurium to the tested antibiotics was mediated by CpxR-P.

**Table 3:** **Susceptibility of ***S. enterica*** serovar Typhimurium to several antibiotics**.

**Strain**	**MICs (μg/mL)**								
	**AMK**	**GEN**	**APR**	**NEO**	**CRO**	**CEF**	**CEQ**	**ENR**	**CIP**
JS	0.5	0.25	2	0.4	0.02	0.32	0.08	0.032	0.016
JSΔ*cpxR*	0.125	0.0625	1	0.1	0.01	0.08	0.04	0.032	0.016
JSΔ*cpxR*/p*cpxR*	0.5	0.25	4	0.4	0.04	0.32	0.16	0.032	0.016
JSΔ*cpx*R/pcpxR^*^	0.125	0.0625	1	0.1	0.01	0.08	0.16	0.032	0.016
JSΔ*acrB*	0.25	0.25	2	0.2	0.01	0.0025	0.02	0.001	0.001
JSΔ*acrB*Δ*cpxR*	0.0625	0.0625	0.5	6.4^▴^	0.01	0.00125	0.02	0.001	0.001
JSΔ*acrB*Δ*cpxR*/p*cpxR*	0.5	0.25	2	25.6^▴^	0.02	0.005	0.08	0.001	0.001
JSΔ*acrB*Δ*cpxR*/p*cpxR*^▴^	0.0625	0.0625	0.5	6.4^▴^	0.01	0.00125	0.04	0.001	0.001

*AMK, Amikacin; GEN, Gentamycin; APR, Apramycin; NEO, Neomycin; CRO, Ceftriaxone; CEF, Ceftiofur; CEQ, Cefquinome; ENR, Enrofloxacin; CIP, Ciprofloxacin*.

▴ *High NEO resistance is present in strains because of the replacement of cpxR gene by kanamycin-resistant gene (ΔcpxR::kan)*.

### Effects of deletion of *acrB* on *cpxR*-mediated multidrug resistance

In susceptible *S. enterica* serovar Typhimurium, the AcrAB efflux pump is constitutively expressed and plays a predominant role in intrinsic and acquired resistance (Mazzariol et al., 2000; Nishino et al., 2006). It has wild substrate spectrum and can capture substrates from the periplasm or the outer leaflet of the cytoplasmic membrane (Yu et al., 2003). Therefore, AcrAB may mask partial function of some efflux pumps located in the cytoplasmic membrane (Hirakawa et al., 2003a,b; Eaves et al., 2004; Nishino et al., 2007). To clarify the role of CpxR in resistance conferred by other efflux pumps, an *acrB* deletion mutant (JSΔ*acrB*) and a double deletion mutant (JSΔ*acrB*Δ*cpxR*) were generated from strain JS. The *cpxR* complementary strain JSΔ*acrB*Δ*cpxR*/p*cpxR* was prepared as described above. The MICs of various antibiotics for strains JSΔ*acrB*, JSΔ*acrB*Δ*cpxR*, and JSΔ*acrB*Δ*cpxR*/p*cpxR* were then determined. As shown in Table 3, strain JSΔ*acrB*Δ*cpxR* showed 2–4-fold decreases in the MICs of GEN, AMK, APR, NEO, and CEF, as compared to strain JSΔ*acrB*, while the complementary strain JSΔ*acrB*Δ*cpxR*/p*cpxR* exhibited 2–8-fold increases in the MICs of GEN, AMK, APR, NEO, CRO, CEF, and CEQ, as compared to strain JSΔ*acrB*Δ*cpxR*. These results revealed that CpxR can modulate resistance of *S. enterica* serovar Typhimurium to aminoglycosides and β-lactams in both *acrB* and Δ*acrB* backgrounds.

### Role of *cpxR* in drug resistance of *S. enterica* serovar typhimurium isolates

To determinate the role of *cpxR* in regulating drug resistance of *S. enterica* serovar Typhimurium isolates, nine *cpxR*-deficient mutants (SH1–9Δ*cpxR*) derived from nine *S. enterica* serovar Typhimurium isolates (SH1–9) were constructed. The MICs for SH1–9 and SH1–9Δ*cpxR* to the above antibiotics were then determined. As shown in Table 4, among the nine *cpxR* deletion strains, all showed 2–4-fold decreases in the MICs of GEN, AMK, APR, and CEF, six revealed 2–4-fold decreases in the MIC of CRO, and six revealed 2–4-fold decreases in the MIC of CEQ, compared with their parental strains. These results indicate that *cpxR* also plays an important role in resistance of *S. enterica* serovar Typhimurium isolates to aminoglycosides and β-lactams.

**Table 4:** **Susceptibilities of ***S. enterica*** serovar Typhimurium isolates to antibiotics after ***cpxR*** were deleted**.

**Strains**	**MICs (μg/mL)**						
	**AMK**	**GEN**	**APR**	**NEO**	**CRO**	**CEF**	**CEQ**
SH1	1.25	1	2	0.8	0.5	4	1
SH1Δ*cpxR*	0.625	0.125	0.5	12.8^▴^	0.5	2	1
SH2	5	32	4	0.4	0.5	4	0.5
SH2Δ*cpxR*	1.25	16	2	6.4^▴^	0.25	2	0.25
SH3	2	0.2	4	0.4	0.1	0.8	0.05
SH3Δ*cpxR*	0.5	0.1	1	6.4^▴^	0.05	0.4	0.05
SH4	1	0.4	2	0.4	0.05	0.8	0.1
SH4Δ*cpxR*	0.5	0.1	1	6.4^▴^	0.05	0.4	0.05
SH5	1	0.4	4	0.4	0.05	0.8	0.1
SH5Δ*cpxR*	0.5	0.2	2	3.2^▴^	0.05	0.4	0.05
SH6	0.4	0.2	8	0.8	0.1	0.2	0.1
SH6Δ*cpxR*	0.1	0.05	2	12.8^▴^	0.05	0.2	0.1
SH7	1.6	12.8	1024	1.6	0.1	1.6	0.2
SH7Δ*cpxR*	0.4	3.2	256	12.8^▴^	0.05	0.8	0.1
SH8	0.4	0.2	4	0.8	0.2	0.4	0.1
SH8Δ*cpxR*	0.2	0.1	2	12.8^▴^	0.05	0.1	0.05
SH9	0.8	0.4	4	0.8	0.1	0.8	0.05
SH9Δ*cpxR*	0.1	0.05	1	12.8^▴^	0.05	0.4	0.025

*AMK, Amikacin; GEN, Gentamycin; APR, Apramycin; NEO, Neomycin; CRO, Ceftriaxone; CEF, Ceftiofur; CEQ, Cefquinome*.

▴ *High NEO resistance is present in strains because of the replacement of cpxR gene by the kanamycin-resistant gene (*Δ*cpxR::kan)*.

### Effects of *cpxR* on the expression levels of a series of MDR-related genes

**Figure 1:**
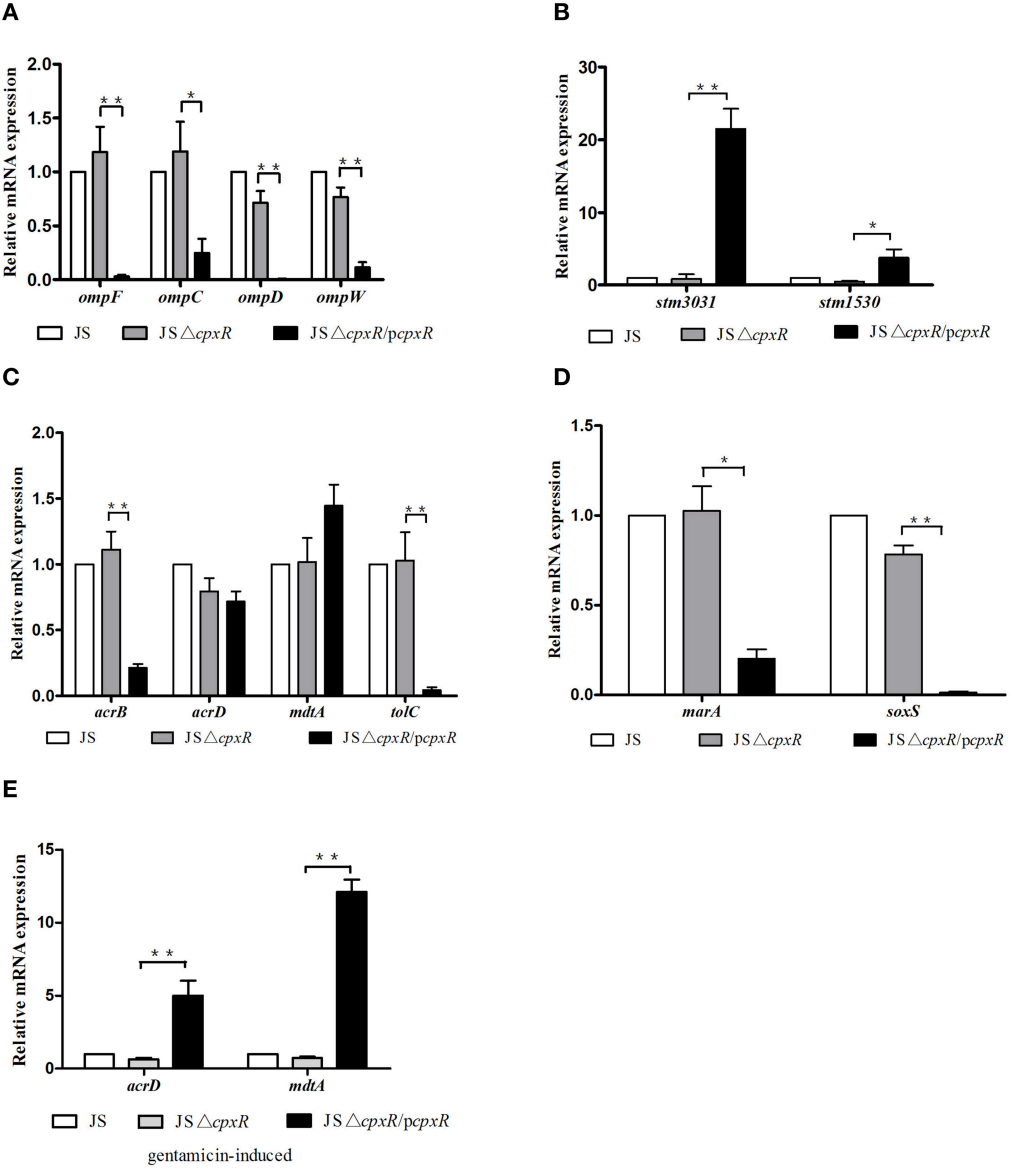
**Relative mRNA expression levels (n-fold) determined by real-time PCR**. The expression level of each mRNA in strain JS represents 1-fold. The expression of the 16S rRNA gene was used as an internal control. Each bar represents the average value of three independent experiments. **(A)** Relative mRNA expression levels of the outer membrane proteins genes *ompF, ompC, ompD*, and *ompW*; **(B)** Relative mRNA expression levels of the outer membrane protein genes *stm3031* and *stm1530*; **(C)** Relative mRNA expression levels of the efflux pumps genes *acrB, acrD, mdtA*, and *tolC*; **(D)** Relative mRNA expression levels of the transcription factor genes *marA* and *soxS*. **(E)** Relative mRNA expression levels of the efflux pump genes *acrD* and *mdtA* in all tested strains induced by GEN to the 15th passage at subinhibitory concentrations. ^*^p < 0.05, ^**^p < 0.01.

In *E. coli*, it has been confirmed that *cpxR* can modulate the expression of the outer membrane proteins OmpF and OmpC (Batchelor et al., 2005), and the transporter MdtABC (Hirakawa et al., 2005). In *S. enterica* serovar Typhimurium, the expression of OmpD, STM3031, and STM1530 plays important roles in *cpxR*-mediated CRO resistance (Hu et al., 2011). In order to determine whether the drug resistance mediated by *cpxR* is due to altered expression levels of MDR-related genes, we detected the relative mRNA expression of a series of MDR-related genes. As shown in Figure 1, JSΔ*cpxR* showed no significant differences in the mRNA expression levels of all tested genes, as compared to strain JS, while the mRNA expression levels of *ompF, ompC, ompW, ompD, acrB, tolC, marA*, and *soxS* genes in strain JSΔ*cpxR*/p*cpxR* were significantly decreased (*p* < 0.01 or *p* < 0.05) relative to strain JSΔ*cpxR* (Figures 1A,C,D) and the mRNA levels of *stm3031* and *stm1530* in strain JSΔ*cpxR*/p*cpxR* were significantly increased (*p* < 0.01 or *p* < 0.05) relative to strain JSΔ*cpxR* (Figure 1B). There were no significant differences in mRNA expression levels of *acrD* and *mdtA* among strains JS, JSΔ*cpxR*, and JSΔ*cpxR*/p*cpxR* (Figure 1C). The expression levels of AcrF in these three strains were all very low and almost undetectable (data not shown). However, after all strains were induced by GEN at subinhibitory concentrations to the 15th passage simultaneously, strain JSΔ*cpxR*/p*cpxR* showed significant (*p* < 0.01) increases in the mRNA expression levels of *mdtA* and *acrD*, as compared to JSΔ*cpxR* (Figure 1E). These results suggest that the overexpression of *cpxR* can downregulate the expression levels of OmpF, OmpC, OmpW, OmpD, AcrB, TolC, MarA, and SoxS and upregulate those of STM3031 and STM1530 in susceptible *S. enterica* serovar Typhimurium strains, and it also upregulated the expression levels of the efflux pumps AcrD and MdtA under the pressure of GEN.

## Discussion

In this study, we analyzed the effect of CpxR on the drug resistance of a susceptible strain and nine clinical isolates of *S. enterica* serovar Typhimurium and found 2- to 4-fold decreases in resistance to aminoglycosides and β-lactams by deletion of *cpxR* (Tables 3, 4). These results are similar to those of previous studies reporting that the overexpression of *cpxR* in *E. coli* caused 2-fold increases in resistance to β-lactams (Hirakawa et al., 2003a), but different from the findings that the *cpxA-cpxR* deleted mutant R200(Δ*cpxAR*) showed more remarkable decreases (>2048-fold) than strain JSΔ*cpxR* in the MIC of CRO, as compared to their parental strain (Hu et al., 2011). Obviously, R200, generated by a multistep resistance selection method, is a CRO-resistant strain. Therefore, we concluded that the influence of *cpxR* on the drug resistance of resistant strains is greater than that of susceptible strains.

It is known that OmpF and OmpC are the most abundant outer membrane proteins of *S. enterica* serovar Typhimurium. Many antimicrobial agents have been found to alter the expression of these proteins. Moreover, it has been confirmed that decreased level of OmpD, and increased levels of STM3031 and STM1530 are associated with *S. enterica* serovar Typhimurium CRO resistance (Hu et al., 2009, 2011). In this study, we found significant reductions in the levels of OmpF, OmpC, OmpD, and OmpW, and significant increases in levels of STM3031 and STM1530 when *cpxR* was reverted to strain JSΔ*cpxR*. Thus, the altered levels of the above-mentioned outer membrane proteins influenced by CpxR may be closely associated with the CpxR-mediated resistance of *S. enterica* serovar Typhimurium to β-lactams.

In Gram-negative bacteria, transporters belonging to the RND family are particularly effective in generating resistance, and MDR often results from the overexpression of multidrug efflux transporters (Grkovic et al., 2002). In this study, before the strains were induced with GEN, CpxR overexpression led to significant reductions in levels of AcrB, TolC, MarA, and SoxS. MarA and SoxS are global regulatory factors (Wall et al., 2009). Once overexpressed, MarA further activates AcrAB/TolC efflux and alters the expression of some membrane proteins (Sulavik et al., 1997). To our knowledge, the influence of CpxR on the mRNA levels of *marA* and *soxS* genes has not been demonstrated. Our results can give two suggestions. One is that the decrease in AcrAB–TolC, mediated by the complementation of *cpxR*, is associated with the decrease of the regulatory factors MarA and SoxS. The other is the expression levels of AcrB and TolC do not play a decisive role in CpxR-mediated resistance of *S. enterica* serovar Typhimurium to aminoglycoside and β-lactams. Our finding that CpxR can influence the susceptibility of *S. enterica* serovar Typhimurium to aminoglycosides and β-lactams in both *acrB* and Δ*acrB* backgrounds also supports the second suggestion. Nevertheless, more studies should be carried out to elucidate the reciprocal relationship among CpxR, outer membrane protein genes, efflux genes, and regulative genes.

In this study, the up-regulatory effect of CpxR on the expression levels of AcrD and MdtA were observed in the GEN-induced strains. Aminoglycoside uptake in Gram-negative bacteria includes three consecutive steps. The first step is an electrostatic interaction between aminoglycosides and the bacteria cell envelope through displacement of Mg^2+^ and Ca^2+^ ions that link adjacent lipopolysaccharide molecules, which damages the bacteria outer membrane and enhances its permeability. The second step is energy-dependent phase I of uptake, which leads to a small quantity of antibiotic molecules transversing the cytoplasmic membrane. The third step is energy-dependent phase II of uptake, in which misfolded proteins are produced due to the binding of incoming antibiotics to the ribosome. Some of these proteins are incorporated in the cytoplasmic membrane leading to the loss of membrane integrity. Therefore, additional quantities of aminoglycosides are transported across the damaged cytoplasmic membrane (Taber et al., 1987). Thus, CpxR may be activated by GEN in the inducing experiment *in vitro*. It has been reported that the promoter regions of *acrD* and *mdtABC* harbor binding sites for the response regulator BaeR (Nishino et al., 2007). CpxR also can bind to the *cpxR* box located in the promoter region of target genes. In common, the consensus *cpxR* box includes a tandem repeated GTAAA sequence that is separated by a 5-bp space (Batchelor et al., 2005). The DNA binding feature of CpxR encouraged us to analyze the promoter region of *acrD* and *mdtA* in the chromosome of *S. enterica* serovar Typhimurium LT2 (accession number: AE006468) for the presence of putative CpxR binding sites. Interestingly, our analysis revealed the presence of two similar sequences located 173 bp (site 1: GTAAA-gaacg-GCAAA) and 106 bp (site2: GTAAA-agcgc-ATGAT) upstream of the *acrD* translational start site, respectively. Among them, site 1 was also found 328 bp upstream of the *kpnEF* translational start site. Furthermore, it has been confirmed that purified CpxR from a strain of *K. Pneumoniae* can directly bind to site 1 (Srinivasan and Rajamohan, 2013). Because CpxR of *K. Pneumoniae* exhibits the highest level of homology to CpxR of *S. enterica* serovar Typhimurium (96%), CpxR of *S. enterica* serovar Typhimurium may directly bind to the promoter region of *acrD*. As we know, AcrD participates in the efflux of aminoglycosides, thus our analysis indicates that CpxR contributes to AcrD-mediated resistance of *S. enterica* serovar Typhimurium to GEN, which belongs to aminoglycosides.

Moreover, in this study, there were no significant differences in the mRNA expression levels of all tested genes in strain JSΔ*cpxR*, as compared to strain JS. It has been demonstrated that histidine kinase (HK) also possess response regulator phosphatase activity, which may ensure that the response regulator remains inactive in the absence of activating signals (Raivio and Silhavy, 1997). Therefore, we think that the response regulator CpxR is always in a pre-stimulated resting state and does not modulate mRNA levels at physiological levels. Biochemical data suggest that CpxR can become phosphorylated by the low-molecular-weight phospho-donor acetyl phosphate and, further, that this form (CpxR-P) has a greater affinity for binding to the promoters of target genes (Pogliano et al., 1997). Similarly, it has been demonstrated that, in the absence of CpxA, CpxR can transcriptionally activate downstream target genes, suggesting that CpxR-P is responsible for transcriptional activation of target genes (Danese et al., 1995; De Wulf and Lin, 2000; Batchelor et al., 2005). In this scenario, we conclude that CpxR overproduction in JSΔ*cpxR* encourages the emergence of CpxR-P, which acts as a modulator of gene expression.

In summary, we have reported the first systematical and extensive study about the role of CpxR in aminoglycoside and β-lactams resistance in both susceptible strains and clinical isolates of *S. enterica* serovar Typhimurium. Our results not only clearly confirmed that CpxR contributes to resistance of *S. enterica* serovar Typhimurium to aminoglycoside and β-lactams but also indicated that the effect of CpxR on the expression levels of MDR-related genes is closely associated with CpxR-mediated resistance of *S. enterica* serovar Typhimurium to aminoglycoside and β-lactams. This is the first time that the effect of CpxR on the expression levels of *marA* and *soxS* genes have been investigated in *S. enterica* serovar Typhimurium. Further studies are obviously required to investigate the reciprocal relationship among CpxR, MDR-related outer membrane protein genes, efflux pump genes and regulative genes including *marA* and *soxS*.

## Author contributions

HH, YS, and GH conceived of the study, and participated in its design and coordination. YG, CM isolated the *S. enterica* Serovar Typhimurium isolates. HH carried out the antibiotics susceptibility testing and molecular biology studies, including gene deletion, construction of expression vector and RT-PCR. HH and YP performed the statistical analysis. HH drafted the manuscript. YS, LY, and GH revised the manuscript.

## Funding

This study was supported by grants from the National Natural Science Foundation of China (31372481) and the Key Research Program of Higher Education of Henan Province (15A230006).

### Conflict of interest statement

The authors declare that the research was conducted in the absence of any commercial or financial relationships that could be construed as a potential conflict of interest.
